# Simultaneous Sinus Lifting and Alveolar Distraction of a Severely Atrophic Posterior Maxilla for Oral Rehabilitation with Dental Implants

**DOI:** 10.1155/2012/471320

**Published:** 2012-06-25

**Authors:** Takahiro Kanno, Masaharu Mitsugi, Jun-Young Paeng, Shintaro Sukegawa, Yoshihiko Furuki, Hiroyuki Ohwada, Yoshiki Nariai, Hiroaki Ishibashi, Hideaki Katsuyama, Joji Sekine

**Affiliations:** ^1^Division of Oral and Maxillofacial Surgery, Kagawa Prefectural Central Hospital, 7608557 Kagawa, Japan; ^2^Department of Oral and Maxillofacial Surgery, Shimane University Faculty of Medicine, 6938501 Izumo, Japan; ^3^OMS Takamatsu, 7600047 Kagawa, Japan; ^4^Department of Oral and Maxillofacial Surgery, Samsung Medical Center, 130710 Seoul, Republic of Korea; ^5^Ohwada Dental Clinic, 1430014 Tokyo, Japan; ^6^MM Dental Clinic, Center of Implant Dentistry, 2200012 Yokohama, Japan

## Abstract

We retrospectively reviewed a new preimplantation regenerative augmentation technique for a severely atrophic posterior maxilla using sinus lifting with simultaneous alveolar distraction, together with long-term oral rehabilitation with implants. We also analyzed the regenerated bone histomorphologically. This study included 25 maxillary sinus sites in 17 patients. The technique consisted of alveolar osteotomy combined with simultaneous sinus lifting. After sufficient sinus lifting, a track-type vertical alveolar distractor was placed. Following a latent period, patient self-distraction was started. After the required augmentation was achieved, the distractor was left in place to allow consolidation. The distractor was then removed, and osseointegrated implants (average of 3.2 implants per sinus site, 80 implants) were placed. Bone for histomorphometric analysis was sampled from six patients and compared with samples collected after sinus lifting alone as controls (*n* = 4). A sufficient alveolus was regenerated, and all patients achieved stable oral rehabilitation. The implant survival rate was 96.3% (77/80) after an average postloading followup of 47.5 months. Good bone regeneration was observed in a morphological study, with no significant difference in the rate of bone formation compared with control samples. This new regenerative technique could be a useful option for a severely atrophic maxilla requiring implant rehabilitation.

## 1. Introduction

Treatment with dental implants has become a new paradigm in oral and maxillofacial reconstruction and rehabilitation after teeth loss with maxillary alveolar resorption. However, appropriate implant positioning can be compromised by insufficient bone volume and the surrounding soft tissue condition of the residual alveolus. Severe atrophy (Class >IV, according to the classification of Cawood and Howell) of a totally or partially edentulous maxilla can pose a major challenge for implant-supported fixed oral rehabilitation [1, 2]. The three-dimensional (3D) centripetal bone resorption pattern of the maxilla, especially when associated with centrifugal resorption of the mandible, can create a relatively unfavorable vertical, transverse, and sagittal intermaxillary relationship, which can further hinder maxillary implants and make implant functional rehabilitation difficult [2, 3]. Thus, the volume of healthy maxillary bone and intermaxillary positioning must be increased and improved, the condition of the bone and surrounding soft tissue must be improved, and the interarch situation must be corrected [1–3].

As maxillary bone resorption with alveolar atrophy varies among patients, different reconstructive surgical techniques, including saddle or veneer onlay bone grafting, maxillary sinus floor elevation with grafting, and various guided bone regeneration techniques, alone or in combination, have been introduced [4, 5]. These augmentation methods can effectively create adequate bone volume for implant sites and lead to successful long-term implant treatment [4–6]. However, most fail to recreate the optimal intermaxillary three-dimensional relationship for correct prosthetic rehabilitation, and there may be a limited amount of bone available for augmentation because of soft tissue coverage and donor site issues [4, 6]. Furthermore, careful attention must be taken to avoid an incorrect crown-to-implant ratio in a prosthesis, a flawed intermaxillary relationship, undesirable peri-implant conditions, and bone resorption due to difficult maintenance [4, 7]. 

For the severely atrophic maxillary alveolus, the advent of Le Fort1 osteotomy with autogenous interpositionnal bone grafting, typically using iliac bone, allows forward and/or downward repositioning of the maxilla. It also provides sufficient bone volume for the insertion of appropriately sized implants in an ideal position, and a better crown-to-implant ratio in the prosthesis. This technique, used with simultaneous implant placement for bone graft stabilization, was first described by Sailer [8]. Various modifications of the technique include horseshoe sandwich osteotomy, unilateral segmental osteotomy with interpositional bone grafting, and procedures designed for mucosal sinus preservation in bone grafting [4, 9, 10].

Alveolar distraction has recently gained acceptance as a predictable preimplant augmentation method for simultaneously regenerating bone and surrounding soft tissue [11, 12]. For an extremely atrophic posterior maxillary region, we have developed a modified technique that combines sinus lifting with simultaneous alveolar distraction, instead of the interpositional bone grafting of the Le Fort1 osteotomy [12]. This technique compensates for the conventional sinus lifting approach used for implant treatment and is less surgically invasive. It allows the regeneration of native bone and soft tissue and provides a controllable distracted alveolar segment for the implant prosthesis, regenerating the augmented vertical dimension. As described in our preliminary technical notes published in 2005 [12] and 2009 [13], the mid-term clinical results for a partially or totally edentulous atrophic maxillary alveolus showed optimal implant rehabilitation. Consequently, we have widened the indications for this technique to include severe atrophy (Class >IV, according to the classification of Cawood and Howell [1]) of a totally or partially edentulous maxilla in patients needing implant-supported fixed oral rehabilitation.

This study retrospectively evaluated the efficacy of our new pre-implant reconstruction technique using sinus lifting with simultaneous alveolar distraction for regenerating a severely atrophic maxilla, with long-term oral rehabilitation with implants, and analyzed the regenerated bone histologically.

## 2. Patients and Methods

**Figure 1:**
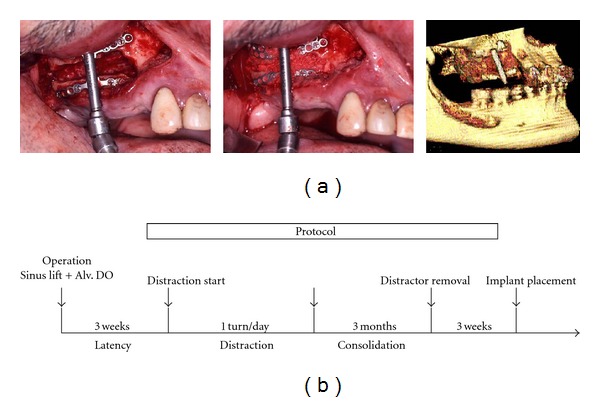
Surgical technique and treatment protocol for simultaneous sinus lifting and alveolar distraction. (a) Intraoperative views and 3D computed tomography of the result; (b) treatment protocol for simultaneous sinus lifting with alveolar distraction and implant placement.

This study included 25 maxillary sinus sites in 17 systemically healthy patients (9 females, 8 males; average age 49.3 years; Tables 1 and 2). All patients had a partially or totally edentulous severely atrophied posterior maxilla, Class IV, V, or VI according to the Cawood/Howell classification [1], with relevant maxillary retrusion, interarch distance with incorrect crown-to-implant ratio, or difficulty in wearing an ordinary dental implant-anchored fixed prosthesis. Thus, these patients were possible candidates for our pre-implant surgical method of sinus lifting with simultaneous alveolar distraction, to accomplish oral implant-anchored prosthetic functional rehabilitation (Figure 1).

All patients gave informed consent before participating in this study. 

The patients were evaluated using radiography, plaster modeling, and computed tomography (CT; Somatom AR SP; Siemens, Erlangen, Germany). An accurate 3D analysis of the planned surgery was performed using SimPlant OMS (Materialise, Leuven, Belgium), which allows precise 3D simulation of the morphologically complex severely atrophic maxillary alveolar ridge with the maxillary sinus. The original residual alveolar bone height was analyzed based on preoperative CT data for the simulated dental implant positions, measuring from the alveolar crest to the floor of the maxillary sinus. The average data for all of the measured simulated implant positions in the atrophic posterior maxilla for each patient were summarized as the preoperative residual bone height (Tables 1 and 2). It also simulates the 3D changes with gradual repositioning following alveolar distraction, considering the intermaxillary relationship with occlusion, as reported previously [14].

Patient exclusion criteria were (1) tobacco abuse (>20 cigarettes/day), (2) renal or liver disease, (3) history of radiography of the head and neck region, (4) history of chemotherapy for a malignant tumor, (5) uncontrolled diabetes, (6) history of bisphosphonate administration, (7) oral mucosal disease such as lichen planus, (8) poor oral hygiene, and (9) noncompliance.

### 2.1. Reconstructive Surgical Procedure

 For patients with a totally edentulous bilateral severely atrophied posterior maxilla needing bilateral sinus lifting with simultaneous total maxillary alveolar distraction, surgery was performed under general anesthesia with nasal endotracheal intubation (Table 1). On the other hand, for patients with a partially edentulous severely atrophic posterior maxilla needing unilateral simultaneous sinus lifting and alveolar distraction, surgery was performed under local anesthesia and intravenous sedation (Table 2). At the start of the operation, patients were given 1 g of cefazolin sodium and 4 mg of betamethasone. Local anesthesia with a vasoconstrictor was used to minimize bleeding in the soft tissue.

**Figure 2:**
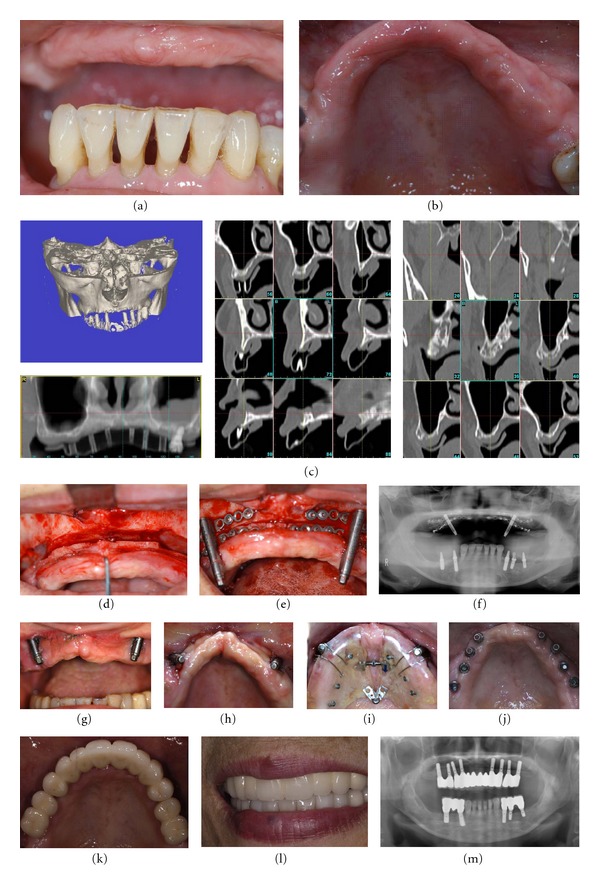
Representative case of bilateral sinus lifting and simultaneous total alveolar distraction for an edentulous severely atrophic maxilla (Case 2, Table 1). (a, b) Preoperative intraoral views; (c) preoperative CT views; (d) intraoperative view. After the end of the bilateral sinus floor elevation and completion of the total alveolar osteotomy: (e)sufficient sinus lifting with an equal-volume mixture of particulate autogenous cancellous bone/*β*-TCP was observed and the bilateral alveolar distractors were set; (f) postoperative panoramic X-ray; (g) after the end of vertical distraction; (h) good vertical distraction was obtained, but the maxillary alveolar arch was very narrow and V-shaped; (i) bilateral alveolar segmental widening with distraction was followed with use of an orthodontic palatal expansion device (the Hyrax device); (j) implant placement at ideal positioning was obtained for dental implant-anchored fixed prosthetic rehabilitation; (k, l) the definite prosthesis was set after 2 years with a provisional restoration during the postloading period for total oral rehabilitation; (m) panoramic X-ray taken 3 years later.

**Figure 3:**
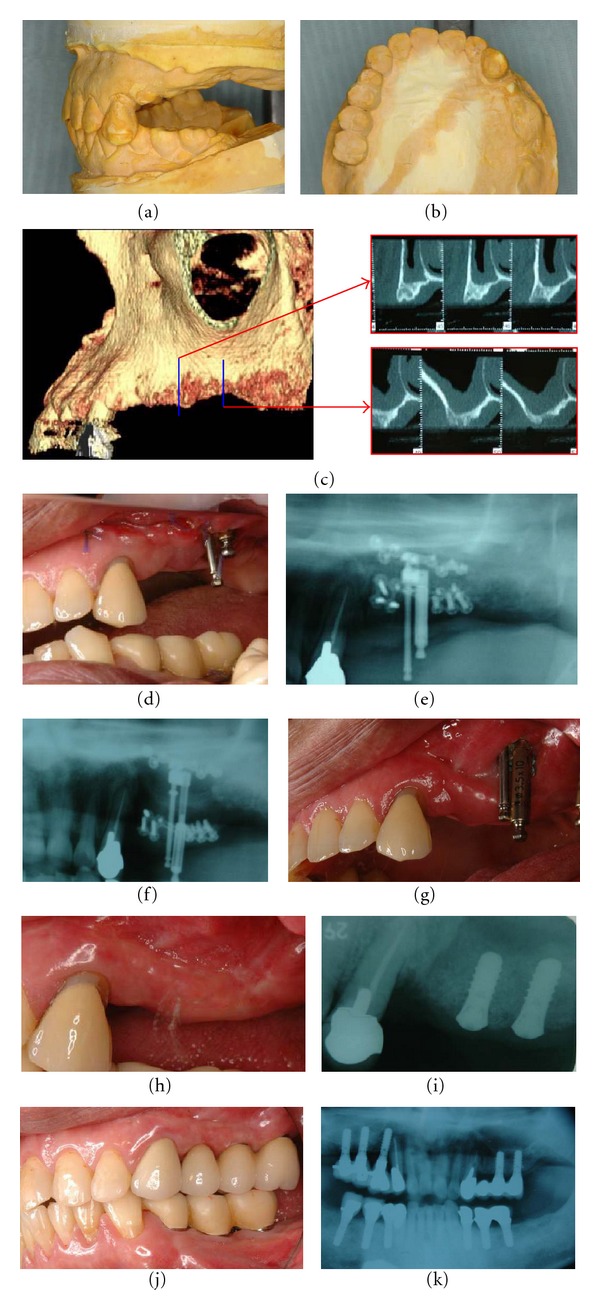
Representative case of unilateral sinus lifting and simultaneous unilateral alveolar distraction for a unilateral partially edentulous, severely atrophic posterior maxilla (Case 5, Table 2). (a, b) Preoperative plaster models of the left posterior maxillary atrophy; (c)preoperative CT views; (d) postoperative intraoral view; (e) postoperative X-ray before activation of distraction with unilateral sinus lifting and simultaneous alveolar distractor setting; (f) postoperative panoramic X-ray after the end of distraction; (g) after the end of vertical distraction; (h) at the time of implant placement and 3 weeks after distractor removal for soft tissue healing; (i) dental X-ray after implant placement; (j)the definite prosthesis was placed after 1 year of provisional restoration as the postloading period; (k) panoramic X-ray taken after 2 years.

A vestibular incision was made extending from the second molar area on one side to the other. A mucoperiosteal flap was reflected, and the alveolar crest and entire lateral maxillary sinus wall and piriform aperture were deflected to perform the alveolar osteotomy. A horizontal alveolar osteotomy for unilateral simultaneous sinus lifting and alveolar distraction was carefully made 3–5 mm from the alveolar crest, which was planned and simulated preoperatively, as described previously [12]. This left ample alveolar bone to fix the plate of the distractor, usually done using a small round bur or Piezosurgery to protect the intact sinus membrane. The sinus membrane was then lifted, taking care to avoid perforating the maxillary sinus. A total alveolar osteotomy for bilateral sinus lifting with simultaneous total maxillary alveolar distraction was completed with a bone saw for bilateral distraction in edentulous cases (Figure 2) [13]. For unilateral cases, a box-shaped window osteotomy of the alveolar bone was made, after the maxillary sinus membrane was carefully lifted (Figure 3) [12]. Confirmation of the reflection and upward lifting was performed, and the palatal side of the alveolus was then completely osteotomized with a bone saw or Piezosurgery to make the transport segment, checking the osteotomy line with a forefinger touching the palatal mucosa. After the transport segment, either a total alveolar osteotomized segment in bilateral cases or a unilateral bow-shaped alveolar osteotomized segment in unilateral cases was prepared, lifting the sinus membrane. An alveolar distractor (KLS Martin, Tuttlingen, Germany; Medartis AG, Basel, Switzerland) (Tables 1 and 2) was positioned with fixation at bilateral molar sites for most total alveolar distraction cases, or a single alveolar distractor was positioned at the central maxilla when sufficient bone remained for distractor setting at the anterior maxillary alveolus (Figure 2). Alternatively, a single distractor adjusted for unilateral distraction cases was set at the sinus-lifted unilateral molar site (Figure 3). After confirming its rigid fixation, the distractor was activated once to widen the window for sufficient maxillary sinus lifting with an equal-volume mixture of particulate autogenous cancellous bone/*β*-TCP particle grafting material (Olympus, Tokyo, Japan; Pentax, Tokyo, Japan) for sinus floor elevation. The distractor was then returned to its initial position. The alveolar bone and maxillary sinus membrane were protected from injury during the operation. The surgical wounds were sutured without tension by relaxing the soft tissue flaps.

Surgery was performed simultaneously on the donor and recipient sites. Bone donor sites were chosen intraorally from the mandibular chin or ramus, or extraorally from the medial portion of tibia cancellous bone, based on the patient's choice (Tables 1 and 2). The cases for bilateral sinus lifting with total alveolar distraction tended to need more bone, and thus the tibia was chosen as the autogenous bone donor site. Patients were hospitalized for 1–3 days after surgery. Intraoperative and postoperative clinical courses were uneventful.

 Following a latent period of 3 weeks, patient self-distraction was started at a slow rate of one turn (0.5 mm) per day (Figure 1). Next, the activation rate was accelerated to two turns (1.0 mm) per day, considering the soft tissue condition. When the required augmentation was achieved, the distractor was left in place for 3 months to ensure bony consolidation. Then, it was removed to allow implant insertion. For some bilateral distraction cases with total maxillary distraction with bilateral alveolar distractors, the bilateral maxillary alveolar segments were distracted palatally due to the directions of the distractors, together with the tight tension of the palatal mucosa (Figure 2). After sufficient vertical distraction, bilateral alveolar segmental widening was performed with an orthodontic palatal expansion device (Hyrax device), so that the bimaxillary relationship and a suitable maxillary arch were acquired (Figure 2).

### 2.2. Dental Implant Surgery

 After the alveolar distractor was removed, soft tissue was allowed to heal for 3-4 weeks before implant surgery (Figure 1). Further instructions included diet, the use of a 0.12% chlorhexidine mouth rinse, and oral health care. Endosseous osseointegrated dental implants (average of 3.2 implants/maxillary sinus site; 80 implants; Straumann ITI, Institute Straumann AG, Basel, Switzerland; Astra Tech, Astra Tech AB, Göteborg, Sweden; Novel Biocare AB, Göteborg, Switzerland), more than 11 mm in length and 4 mm in platform width, were placed in optimized positions applying a computer-guided surgical template using SimPlant OMS, after consultation with prosthodontists and dental implantologists, who had consulted with us regarding the pre-implant bone augmentation reconstructive surgery. The repeated CT data were further analyzed to measure the regenerated alveoli obtained before implant placement. The implants were supplied with cover screws in a two-stage procedure and left to heal for 4–6 months before secondary abutment connection surgery. All patients had dental implant-anchored fixed prosthetic rehabilitation with the cement-retained prosthetic fixation method.

### 2.3. Bone Histomorphometric Analysis

Using a 2 mm trephine bur, regenerated bone was sampled with informed consent from six patients who underwent bone graft-sinus lifting and alveolar distraction, from the simulated site of the first molar at the time of implant placement. As a control, bone was sampled from four other patients who underwent sinus lifting only as a pre-implant augmentation procedure for two-stage implant placement using the same sinus lifting materials (equal-volume mixture of particulate autogenous cancellous bone/*β*-TCP) with the same bone-healing period postoperatively in the same simulated position of the first molar for implant placement with a very similar residual bone volume for the atrophic posterior maxilla. Each cylindrical specimen acquired with the trephine biopsy was 2 mm in diameter and at least 10 mm in length.

Briefly, the bone specimens were fixed in 10% formalin for 24 h and decalcified in Calci-Clear Rapid (National Diagnostics, Atlanta, GA, USA) for 12 h. The tissues were rinsed in flowing water, treated with a Hypercenter XP tissue processor (Shandon, Cheshire, UK), embedded in paraffin, cut into 4 *μ*m sections, and stained with H&E or toluidine blue. The prepared specimens were observed under a light microscope (Olympus) through a 20× objective. The bone samples were evaluated for bone volume and mineralization of woven bone volume at bone histomorphometry institutes (Kyodo Byori, Hyogo, Japan and Ito Bone Histomorphometry Institute).

### 2.4. Statistical Analysis

 Statistical analyses were performed with StatView 5.0 (SAS Institute, Cary, NC, USA). Student's *t*-test was used to compare bone regeneration and new bone formation between the study (*n* = 6) and control groups (*n* = 4). Statistical significance was defined as *P* < 0.05 (*P < 0.05, **P < 0.01).

## 3. Results

The preimplantation augmentation technique using sinus lifting with simultaneous alveolar distraction for regenerating the maxilla was successful after an uneventful postoperative course. No surgical morbidity, infection, dental or gingival injury, or avascular necrosis occurred.

Sufficient sinus floor elevation together with vertical bone augmentation improved the vertical maxilla-mandibular dimension and created an intermaxillary 3D relationship appropriate for correct prosthetic rehabilitation and an improved implant-crown ratio, compared with the preoperative condition. New bone formation with effective vertical and labial-buccal augmentation was observed radiologically after a consolidation period. At the time of implant placement, the regenerated alveoli were analyzed precisely using CT data for the ideal implant positional simulation to quantify the vertical augmentation measuring from the alveolar crest to the floor of the maxillary sinus once again in each patient. The average alveolar bone height augmented for implant placement was 13.7 (range 11.7–15.3) mm for bilateral cases (Table 1) and 12.9 (range 11.7–14.1) mm for unilateral cases (Table 2) from the original severely atrophic posterior maxilla.

Distraction was required for activation to maximum lengths of 10 or 15 mm. A horizontal widening technique was also required in three of eight bilateral distraction cases; bilateral maxillary molar distraction was performed due to the narrowed bilateral alveolus caused after the end of distraction (Figure 2). Soon after the end of distraction, the patients wore palatal expansion orthodontic devices for maxillary arch form correction. The duration of active distraction was approximately 3-4 weeks. By the time of implant placement, the overcorrected distracted alveolus was reduced to some extent, despite maximum overcorrection. After allowing 3 weeks for soft tissue healing, 80 endosseous osseointegrated dental implants (3.2 implants/site), more than 11 mm in length and 4 mm in platform width, were placed in optimized positions using a surgical guide, which was analyzed with SimPlant OMS. All patients achieved stable, functional oral rehabilitation with dental implant placement in the regenerated alveolus.

Regarding the implant survival rate, of the 80 inserted endosseous dental implants (Tables 1 and 2), three failed in three patients (one bilateral and two unilateral distraction cases). They were all early failures, with implant loss within the first year after placement. One bilateral case and one unilateral case with failed implants underwent supplementary insertions; the other failed implant inserted at the most distal molar site in a unilateral case was not reinserted. Consequently, the total implant survival rate was 96.3% (77/80) after an average postloading follow-up period of 47.5 months. The implant-anchored fixed bridge stability rate was 100% because all of the patients were still wearing their definite prosthetic restoration at the time when this clinical study was retrospectively reviewed.

**Figure 4:**
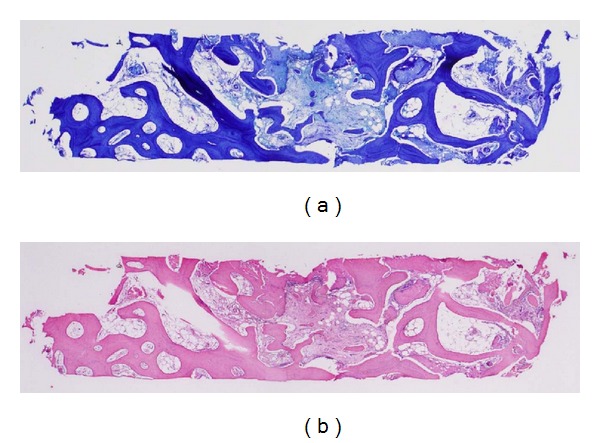
Good bone regeneration was observed, with an average mature bone formation rate of 36.3 ± 16.5%. (a) Stained with toluidine blue; (b) stained with H&E.

Regarding the bone biopsy histomorphometric study, good bone regeneration was observed (Figure 4). An average mature bone formation rate (BV/TV) of 36.3 ± 16.5% (*n *= 6) was confirmed in the bone morphological study at the time of implant placement in the six patients who gave informed consent, with no significant difference from the control samples (39.3 ± 10.4%; *n *= 4) at the same simulated site of the first molar area after an equal bone healing period for consolidation (*P* > 0.05) (Table 3). The bone histomorphometric results regarding new bone formation and maturation were essentially the same between the distracted area of the cases and the control areas at the time of implant placement, which were lifted and not distracted, showing that well-formed new bone to promote good initial stability for dental implant placement was present at the distraction sites with sinus lifting. 

## 4. Discussion

The clinical efficacy of our pre-implant reconstruction technique using sinus lifting with simultaneous alveolar distraction changed the contour of the severely atrophic resorbed maxillary alveolus, regenerating sufficient bone on both the alveolar side and inside the maxillary sinus [12, 13].

As mentioned above, 3D computer simulation allows surgeons to perform virtual surgery and create 3D predictions of patient outcomes. We were able to simulate the entire process of alveolar reconstruction with our technique, including alveolar distraction, the shape of the maxillary sinuses for determining the osteotomy line, the amount of sinus lifting, and the subsequent oral implant placement with parameters that included realistic transport segment lengths, angulation, and prosthesis sizes with easy selection from a very wide variety of preset modules [13, 14]. In the future, one may be able to customize prebent plates for the distractor device preoperatively in cases of complicated reconstruction of a severely atrophic maxilla using distractors.

For dental-implant-retained oral rehabilitation of the severely atrophic maxillary alveolus, the advent of the Le Fort1 osteotomy with autogenous interpositional bone grafting, typically using iliac bone, allows forward and downward repositioning of the maxilla [4, 9, 10]. Bell et al. [15] were the first to describe a Le Fort1 osteotomy with interpositional bone grafts in edentulous patients to improve the positioning of the resorbed maxilla and restore the intermaxillary relationship. This is because, in the severely atrophic maxilla, creating a dental implant site by merely increasing the bony volume will lead to unfavorable loading of the prosthesis and impaired aesthetic and functional results [4, 15]. The conventional Le Fort1 osteotomy with interpositioning of bone grafts for dental implants was first described by Sailer [8] as a one-stage operation, and it was later modified by Cawood et al. [16] as a two-stage procedure. The insertion of the endosseous implants was delayed until the bone grafts had been revascularized. The delayed placement of the dental implants resulted in less risk of their loss and made the use of a template possible [16]. Furthermore, this effective interpositional bone graft with a Le Fort1 level osteotomy was applied to a segmental Le Fort1 osteotomy with bone grafting in the unilateral severely atrophied maxilla based on the ideal movement of the unilateral posterior maxilla, followed by delayed implant placement for ideal oral rehabilitation by Pelo et al. [9].

Our original pre-implant reconstruction technique using sinus lifting with simultaneous alveolar distraction was based on and drastically modified from conventional pre-implant augmentation Le Fort1 osteotomy and interpositional bone grafting [13]. Our osteotomy line was not at the Le Fort1 level, but was an alveolar osteotomy far below this level [11, 12]. This is less invasive and yet sufficient for performing simultaneous sinus lifting with an equal-volume mixture of particulate autogenous cancellous bone/*β*-TCP and placing the distractor [12]. In addition, the secure protection of the surrounding mucoperiosteum, which promotes osteogenic cell and active bone formation with a broad vascular network, plays a role in ensuring nutrition for the healing surgical wound, bone formation, and bone regeneration for simultaneous sinus lifting with alveolar distraction [11, 14]. The closest strategy to our technique is controlling the osteotomized alveolar segment using vertical alveolar distractors with gradual distraction, while managing the direction and amount of distraction. This is unlike the intraoperative critical determination of the 3D positioning of the Le Fort1 segment after osteotomy, which was based solely on the surgeon's experience. Moreover, the volume and position of the interpositioned bone graft are usually limited to some extent due to both bone and soft tissue obstacles [8–10].

Nevertheless, although the simulated distraction was less than the maximum amount, all of the patients needed distraction of 10–15 mm, which would allow compensation for distraction loss and bone loss [11, 14]. Furthermore, in several cases using bilateral buccal alveolar distractors with simultaneous sinus lifting, the bilateral segments distracted well, but the distracted arch was narrowed markedly due to the direction of distraction and the atrophic shape of the residual posterior maxillary alveolus [13]. This was attributable to the resistance of the palatal mucosa and lip support of the functional orbicularis oris muscle [13, 14]. Consequently, these patients needed additional maxillary arch widening distraction control using a Hyrax orthodontic device following vertical distraction, following the “floating bone concept” described by Hoffmeister and Wangerin [17]. This 3D distraction method overcame the arch discrepancy problem for ideal implant positioning, which would not be true of the complete controllability of this distraction technique and contrary to the actual preoperative computer simulation [13]. Furthermore, for cases of unilateral distraction, although improvements in distraction systems are still needed, as evidenced by reports describing major complications, including undesirable palatal-lingual inclination of the transport segments, an undesirable inclination leaning palatally was sometimes seen during the activation period. Therefore, a careful check of the distraction vector with manual manipulation for correction is needed to achieve accurate vector control of the transport segments, together with the use of a fixed or removable prosthodontic to guide the direction of distraction for successful preimplant augmentation, as described previously [14, 18]. This tendency toward problematic palatal inclination was improved to some extent by using bidirectional alveolar distraction systems for the latter cases, as described previously as a floating alveolar segmental control, which could eliminate transport segment displacement without the need for burdensome oral rehabilitation appliances [14, 17, 18]. Furthermore, this segmental postoperative controllability should be effective compared to the one-stage determination of Le Fort1 osteotomy with interpositional bone grafting. We modified the alveolar distraction technique for a severely atrophied posterior maxilla, based on the original idea of Ilizarov [19] that distraction osteogenesis involves the regeneration of bone and surrounding soft tissues through gradual traction between two surgically separated fragments fixed to a mechanical device [20, 21].

The possibility of osteoregeneration using distraction in the posterior maxillary sinus area seemed less likely compared with the mandible or anterior maxilla because of less recruitment of osteoregenerative osteoblasts from the bone marrow, less cancellous bone volume, and the presence of the pneumatized maxillary sinus and sinus membrane is problematic. In 2004, Boyne and Herford [22] first described new bone formation using alveolar distraction in the posterior maxillary sinus area, in an animal study of three adult baboons (*Papio anubis*). This study reflected the clinical difficulty of implant restoration with a thin margin of crestal maxillary alveolar bone attached to the sinus membrane. After placing the distractor against the antral and nasal floors without bone grafting, and allowing a latency period of 7 days, it was activated at a rate of 1 mm/day to obtain 10 mm of lengthening. Twenty weeks after the completion of distraction, specimens of the atrophic maxilla showed significant bone formation [22]. Consequently, bone regeneration is possible in very small segments of the atrophic posterior maxilla using distraction osteogenesis [22]. However, any clinical protocol should include implant placement after bone formation, and no similar animal study has been reported.

Therefore, we hypothesized that a slightly longer latency period would be needed for sufficient distracted bone regeneration in this difficult situation in the atrophic posterior maxilla [13, 14]. In addition, we performed simultaneous autogenous bone grafting after sufficient careful sinus lifting of the membrane, as we believed that autogenous cortical cancellous bone grafting would not only elevate the maxillary sinus floor to make space, but also reliably induce bone formation, with osteoblast recruitment from marrow stem cells and living osteoblasts [21, 23, 24]. Furthermore, we hypothesized that 2-3 weeks would be needed to retain the grafted materials on the lifted maxillary sinuses [12–14]. Without this retention time for the grafted materials, the lifted sinus material would be distracted with the transport segment using the conventional waiting period of 1 week [12, 13]. Therefore, we waited 3 weeks to allow bony retention of the grafted sinus materials and complete soft tissue healing of the surgical site. With this longer waiting period, we did not experience undesirable bony union making it impossible to activate distraction, breakage of the device, or the loss of fixation screws [14, 21, 23]. 

Our histomorphometric study showed good bone regeneration with an average mature bone formation rate (BV/TV) of 36.3%. This rate was similar to Szabó et al.'s [25] report of 38.3% with autogenous cancellous bone grafting versus 36.4% with *β*-TCP particles alone after 6 months. Schwartz et al. [26] reported a 17% bone formation rate using autogenous chin bone with *β*-TCP particles for sinus floor augmentation after 6 months, and a 21% new bone formation rate with a freeze-dried bone allograft and hyaluronic acid for sinus lifting augmentation after 8 months. As bone maturation with good bone regeneration was observed, with an average mature bone formation rate of 36.3%, which did not differ significantly from the 39.3% in the controls with sinus lifting alone for pre-implant augmentation surgery under similar atrophic conditions, the equal-volume mixture of particulate autogenous cancellous bone/*β*-TCP seems to be an ideal graft material, accelerating bone formation in sinus augmentation before implant placement [25–28].

Our clinical results were good, with good mature bone formation and an implant survival rate of 96.3% (77/80 implants) after an average postloading followup of 47.5months. This survival rate is in accord with that of the conventional Le Fort1 technique with interpositional bone grafting with modifications, as described in many reports [9, 10, 29, 30]. The implants used in our study were from different manufacturers and were more than 11 mm in length and had platforms wider than 4 mm. The implants were chosen by the patients' dentists, implantologists, and prosthodontists, and the choice did not seem to influence the survival with our method.

We performed a buccal osteotomy for both total alveolar osteotomy with bilateral sinus lifting and partial box-shaped osteotomy for unilateral sinus lifting with a round burr [12, 13]. Subsequently, we used Piezosurgery initially for the posterior buccal part, being careful not to damage the sinus membrane. Then, the alveolar osteotomy was made with a bone saw or Piezosurgery. Piezosurgery could be very useful for maxillofacial bone surgery to prevent soft tissue damage. Although we were unable to prevent small tears, no patients developed entrapment cysts of the maxillary epithelium or chronic sinusitis.

The main criticism of our study might be the absence of a control group with only sinus lifting for sinus floor elevation without bone grafting with simultaneous alveolar distraction for both bilateral and unilateral cases, as bone regeneration between the sinus membrane and distracted alveolus might occur, as shown in Boyne's animal study [22]. Another major disadvantage of this idea could be simultaneous autogenous bone grafting for sinus lift [12, 13]. Although we believe that simultaneous autogenous bone grafting could play a great role in bone regeneration because it provides large numbers of osteogenic cells for more efficient bone formation at the site of distraction osteogenesis, it requires patients to undergo a second surgical procedure, with harvesting done intraorally (mandibular chin or ramus) or extraorally (tibia) [23]. Fortunately, we did not experience any complications at the donor site. The amount of autograft required could be minimized by combining it with an equal amount of *β*-TCP particles. A point reached in this technique could be how much the simultaneous bone grafting for vertical distraction could contribute to the original distraction technique without sinus lifting alone, or a Le Fort1 internal bone graft at the one-stage surgery exceeding the discomfort of the secondary surgical access for bone harvesting [23, 29, 30].

Recently, many techniques for accelerating bone regeneration during distraction have been introduced, including growth factors and bone morphogenetic proteins [20, 23, 24, 28]. These primarily induce the host tissue to increase the number of osteoblasts, thereby promoting osteogenesis [24, 25]. However, providing viable osteoblasts or preosteoblastic cells via particulate bone grafting might accelerate the osteoregenerative process, as reported here for sinus lifting [13, 23]. Furthermore, the preserved periosteum around the alveolus could play a major role in this alveolar distraction technique. The periosteum contains sufficient osteochondrogenic progenitor cells, which have two potential roles in regeneration: (1) they proliferate and differentiate to form new bone or cartilage and (2) release osteoinductive factors to recruit and activate osteoprogenitor cells from the host [14, 19, 23, 24, 28]. In addition, the straining force produced in the distraction technique may initiate the differentiation of periosteal cells into osteogenic cells, inducing the process of bone regeneration, as described previously [19, 21, 23, 24]. Consequently, conserving the periosteum during surgery may be very important to obtain a successful result using this pre-implant technique [14, 25]. Additional clinical studies are needed to determine the predictability of the regenerative outcomes associated with alveolar distraction osteogenesis with simultaneous autogenous bone grafting.

In conclusion, alveolar distraction is an attractive treatment option for increasing the amount of bone and surrounding soft tissues. Combining it with simultaneous sinus lifting is a useful technique for patients with a severely atrophic maxilla requiring dental implant rehabilitation. Satisfactory long-term implant survival for oral rehabilitation was realized.

## Figures and Tables

**Table 1:** Patient profiles and review (*n* = 8) of bilateral sinus lifting and simultaneous total alveolar distraction for the edentulous severely atrophic maxilla. Bilateral sinus lifting + Total Alv. DO for edentulous patients.

Case	Age (year)	Sex	Alveolar distractor	Donor site	Preoperative residual bone height (mm)	Alveolar bone height at implant placement (mm)	Biopsy	Implant	Postloading (months)
1	43	F	Martin Track 1.5 10 mm	Chin	3.2	14.2	−	6	59
2	69	F	Martin Track 1.5 10 mm	Chin	2.6	13.6	+	8	50
3	50	F	Medartis V2 10 mm	Tibia	3.8	14.5	−	6	48
4	34	M	Medartis V2 15 mm	Tibia	2.9	13.1	−	6	47
5	55	F	Martin Track 1.5 10 mm	Tibia	3.2	11.7	+	8	47
6	54	M	Medartis V2 10 mm	Tibia	2.6	13.9	+	5	48
7	50	F	Medartis V2 10 mm	Tibia	3.5	15.3	−	6	38
8	40	M	Medartis V2 10 mm	Tibia	2.4	12.9	−	6	37

	49.4				3.0	13.7		6.4	46.8

**Table 2:** Patient profiles and review (*n* = 9) for unilateral sinus lifting and simultaneous unilateral alveolar distraction for the unilateral partially edentulous severely atrophic posterior maxilla. Unilateral sinus lifting + Alv. DO for partially edentulous patients.

Case	Age (year)	Sex	Alveolar distractor	Donor site	Preoperative residual bone height (mm)	Alveolar bone height at implant placement (mm)	Biopsy	Implant	Postloading (months)
1	16	M	Martin Track 1.0 9 mm	Ramus	3.8	12.5	−	3	68
2	52	F	Martin Track 1.0 12 mm	Chin	3.7	14.1	−	4	64
3	62	M	Martin Track 1.0 15 mm	Ramus	2.4	12.2	−	4	62
4	48	F	Medartis V2 10 mm	Ramus	2.5	13.9	+	4	48
5	52	M	Medartis V2 10 mm	Ramus	3.5	11.9	−	2	40
6	51	M	Medartis V2 10 mm	Ramus	2.8	11.7	−	3	39
7	58	F	Medartis V2 10 mm	Tibia	2.6	13.1	+	3	38
8	55	M	Martin Track 1.0 15 mm	Ramus	3.6	13.1	−	3	38
9	49	F	Martin Track 1.0 12 mm	Tibia	3.7	13.9	+	3	37

	49.2				3.2	12.9		3.2	48.2

**Table 3:** The bone histomorphometric results for new bone formation and maturation were compared between the study group with alveolar distraction and simultaneous sinus lifting with bone grafting and a control group with sinus lifting only with the same bone graft at the time of implant placement in the simulated first molar area. Student's *t*-test was used to compare bone regeneration and new bone formation between the study group (*n* = 6) and control group (*n* = 4). Statistical significance was defined as *P* < 0.05.

	Age	TV	BV	BV/TV
	(Year)	(*μ*m^2^)	(*μ*m^2^)	(%)
Sinus + Alv. DO (*n* = 6)	54.8	2859626.1	995394.1	36.3
Control (*n* = 4) (sinus lift)	55.3	2823053.3	1138133.8	39.3
*P* (*t*-test)	0.9228	0.9106	0.6207	0.7789
